# Education Research: Predictors of Resident Physician Comfort With Individuals With Intellectual and Developmental Disabilities: A Cross-sectional Study

**DOI:** 10.1212/NE9.0000000000200045

**Published:** 2023-01-05

**Authors:** Hannah F. Johnson Shapiro, Julia S. Frueh, Madeline Chiujdea, Stefan Sillau, Jessica Solomon Sanders

**Affiliations:** From the Department of Neurology (H.F.J.S., J.S.F., M.C.), Boston Children's Hospital, MA; and Department of Neurology (S.S., J.S.S.), and Department of Pediatrics (J.S.S.), University of Colorado School of Medicine, Aurora.

## Abstract

**Background and Objectives:**

Individuals with intellectual and/or developmental disabilities (IDD) experience worse health outcomes compared with peers without a disability partly due to difficulties accessing age-appropriate health care. Provider discomfort with interacting and caring for individuals with IDD is a primary barrier to accessing care. The objectives of this study were to describe resident physicians' education, experiences, and comfort levels regarding individuals with IDD and to identify predictors of higher comfort levels with this patient population.

**Methods:**

In this cross-sectional study, we surveyed medical trainees in 7 residency programs in Boston, Massachusetts on their education, experiences, and comfort levels regarding individuals with IDD. The comfort level was assessed directly on a 6-point Likert scale. The relationship between comfort regarding people with IDD and several candidate explanatory variables was explored with Spearman and partial Spearman correlations (*r*_s_).

**Results:**

The estimated survey response rate was 49%. Of 423 resident physicians included in the study, 96% reported they had treated a patient with IDD, while only 25% reported having formal education on caring for this population. On a scale of 1–6, with higher numbers corresponding to greater comfort, the mean comfort level treating individuals with IDD was 3.73 (CI 3.61–3.85). In bivariant analyses, the amount of prior experience with people with IDD had a moderate, positive correlation with increased comfort levels treating individuals with IDD (*r*_s_ = 0.42, *p* < 0.01). The following characteristics had a weak, positive correlation with increased comfort levels: training in a pediatric-focused residency specialty (*r*_s_ = 0.18, *p* < 0.01), number of hours of formal education on caring for people with IDD (*r*_s_ = 0.15, *p* < 0.01), and age (*r*_s_ = 0.12, *p* = 0.03). Only the amount of prior experience with this patient population remained positively correlated with higher comfort levels when the other variables were controlled for (*r*_s_ = 0.38, *p* < 0.01).

**Discussion:**

Prior experience with individuals with IDD predicted higher comfort levels with this population. This study supports the need for increased opportunities for medical trainees to engage with people with IDD to improve resident physicians' comfort caring for this patient population.

## Introduction

An intellectual disability is defined as a disability that causes significant limitations in both intellectual functioning and adaptive behavior skills, for example, independent living skills. Intellectual disability is a type of developmental disability. A developmental disability refers to a cognitive and/or physical disability that appears before the age of 22 years. Some developmental disabilities are largely physical issues, such as cerebral palsy. Others may include both physical and intellectual disability, such as Down syndrome or autism spectrum disorder. Individuals with intellectual and/or developmental disabilities (IDD) experience worse health outcomes compared with peers without a disability.^1^ This is due in part to difficulties accessing high-quality, age-appropriate health care.^2^ One of the most vulnerable periods for health deterioration in this population is the transition from pediatric to adult healthcare settings. Unfortunately, youth with IDD have significant trouble making this transition.^3-8^ One of the main barriers to individuals with IDD accessing appropriate and effective health care is clinician discomfort with this population.^9,10^

Clinician discomfort caring for individuals with IDD is partially due to a lack of education regarding this patient population. Although more efforts have been made recently to incorporate disability-related content into undergraduate and graduate medical training, many physicians still do not receive adequate education about caring for people with IDD.^11-13^ Pilot data suggest that educational initiatives including didactics, interactive videos, and simulated patient experiences can improve trainee comfort with individuals with IDD.^14-17^ However, there is a paucity of empirical data describing what factors, if any, are associated with increased resident comfort levels in caring for individuals with IDD. In addition, studies examining resident physicians' perceived barriers to caring for individuals with IDD and resident preferences for increasing their comfort levels in caring for this population are lacking.

The objectives of this study were to describe resident physicians' education, experiences, and comfort levels caring for individuals with IDD and to identify predictors of higher comfort levels. The secondary outcomes are to identify residents' perceived barriers to caring for this population and their preferences for strategies to increase their comfort levels.

We hypothesized that residents from pediatric-focused residency programs would be more comfortable with patients with IDD, given these conditions begin in childhood and therefore would be the best predictor of increased comfort levels in our study.

## Methods

A cross-sectional study was designed using an online survey for data collection. The survey was developed in consultation with the Institutional Centers for Clinical and Translational Research at Boston Children's Hospital and included the validated Interaction with Disabled Persons (IDP) scale.^18^ We pilot tested our survey using cognitive interviewing with 4 resident physicians. The survey was then reviewed by residency program directors and resident representatives in each specialty surveyed. Finally, experts in cognitive behavioral neurology and care transition reviewed the survey. We opted to create our own scale for multiple reasons. First, the IDP scale is not specific to persons with IDD, rather any disability. Second, we were interested in measuring physician comfort caring for this population which is not captured in the IDP scale.

### Participants and Setting

Participants included resident physicians from 6 Harvard University–affiliated programs and 1 Tufts University–affiliated program. Two of the programs were in adult neurology, 2 were in internal medicine, 1 was in family medicine, 1 was in child neurology, and 1 was in general pediatrics. The survey was conducted between September and December 2019 and again between October 2020 and March 2021. This survey was part of a conference series designed to increase resident physician comfort caring for individuals with IDD through 1-hour interactive sessions or 1-hour virtual didactic lectures. The results used in this study are from the preintervention surveys collected in both academic years. Because this survey was part of a local educational conference series, we were limited to survey distribution in the Boston area. We targeted general medicine and neurology because these specialties frequently care for this patient population. Beyond this, the study population was a convenience sample.

### Data Management and Survey Distribution

All survey data were managed using Research Electronic Data Capture (REDCap) electronic data capture tools hosted at Boston Children's Hospital. Surveys were distributed in 3 ways to ensure all residents in each program received the survey in an attempt to decrease selection bias and maximize response rate. (1) Chief residents provided the email addresses of all residents currently in their residency training program. Email addresses were then entered directly into REDCap, and surveys were sent through REDCap. (2) Some residency programs did not wish to share the resident email addresses and instead opted to distribute the survey through a REDCap public link sent to an internal listserv that included all current residents in their program. (3) If a resident attended the interactive conference session in the 2019–2020 academic year and did not complete a presession survey through online REDCap, we provided a paper version of the survey that was then manually entered into the REDcap database.

### Variables and Measurement

Participant age, sex, residency specialty, and level of postgraduate year (PGY) training were collected. Participants were asked how many hours of formal education about individuals with IDD they received during their training (0, 1–3, 4–6, 7–9, 10+ hours). Participants were asked how much experience they had with individuals with IDD using a 4-point scale (1 “none,” 2 “a little,” 3 “some,” 4 “much”). Using yes/no questions, participants were asked about different types of experiences they had involving interactions with persons with IDD (10 different experiences were assessed and open-ended responses).

Comfort was assessed directly, on a scale of 1–6, with no neutral point: 1 “very uncomfortable,” 2 “somewhat uncomfortable,” 3 “a little uncomfortable,” 4 “a little comfortable,” 5 “somewhat comfortable,” and 6 “very comfortable.” Comfort was also assessed using the validated IDP scale which is a validated tool “designed to measure emotions, motivations, and reactions that underlie negative attitudes associated with discomfort that some people experience in actual or anticipated social interaction with a person with a disability.”^19,20^ A higher total score on the IDP scale indicates greater discomfort in interactions.

We evaluated the perceived barriers that residents felt affected their ability to treat patients with IDD. The survey listed 14 example barriers, and the residents rated the effect of each on a scale of 1 through 4: 1 “not at all,” 2 “a little,” 3 “somewhat,” and 4 “very much.” We assessed 6 potential interventions that would increase resident comfort. Using a similar 4-point scale, residents were asked to rate how much each intervention would improve their comfort treating patients with IDD. Open-ended responses for both barriers and interventions were also collected.

### Survey Inclusion

Surveys were included for all residents who provided their PGY level and specialty information, and rated their comfort levels with individuals with IDD. If there were 2 surveys completed from the same participant, the most complete survey was included in the analysis. If both entries were complete, then we included the earliest response.

### Survey Response Rate

Because the survey was sent to nonresident participants (i.e., faculty and administrators) through internal listservs, we used the total number of residents in each program provided by the chief residents to determine the denominator in our response rate.

### Residency Program Grouping

We grouped responses from residents in adult-focused programs and pediatric-focused programs for a number of our analyses because this was a prespecified hypothesis. Adult-focused residencies included internal medicine and adult neurology. Pediatric-focused residencies included general pediatrics, child neurology, medicine-pediatrics, and family medicine. Medicine-pediatrics and family medicine were included in the pediatric-focused group because these programs have substantial exposure to pediatrics during their training.

### Statistical Analysis

Continuous variables and Likert scales were summarized with means and were compared between pediatric-focused and adult-focused residency programs with 2 sample *t* tests. To estimate the effect size differences between means, Cohen *d* was used. Some ordinal measures were summarized with medians and compared with the Wilcoxon rank sum (Mann-Whitney) test. Categorical variables were summarized with frequencies and percentages and compared with the χ^2^/Fisher exact test of association. The relationship between comfort interacting and treating people with IDD and several candidate explanatory variables was explored with Spearman and partial Spearman correlation (*r*_s_). In the latter, the relationship between comfort levels and each of the explanatory variables was assessed, while all other variables were controlled for. We performed a subgroup analysis for the adult-focused residents, excluding pediatric-focused residents, as part of this. Strength of correlation (*r*_s_) was rated as follows: 0.0–0.1 (very weak to negligible correlation), 0.1–0.2 (weak, low correlation), 0.2–0.5 (moderate correlation), 0.5–0.8 (strong, high correlation), and 0.8–1.0 (very strong correlation). Correlations that were at least moderate (*r*_s_ ≥ 0.2) were considered educationally meaningful. All tests were 2-sided at a univariate alpha level of 0.05, unless otherwise stated. As the study was primarily exploratory, multiple testing adjustments were not applied. Statistical calculations were performed in SAS 9.4.

### Standard Protocol Approvals, Registrations, and Patient Consents

The Boston Children's Hospital Institutional Review Board determined that this survey-based study was exempt under 45 CFR 46.104(d)(2) from human subjects' review.

### Data Availability

Anonymized data not published within this article will be made available by request from any qualified investigator.

## Results

Approximately 873 surveys were sent to residents, 483 surveys were completed, and 423 surveys met inclusion criteria, resulting in an estimated response rate of 49%.

### Descriptive Data and Comfort Levels

Demographic information for included participants is outlined in Table 1. Across all residents surveyed, 96% reported they had treated a patient with IDD, while only 25% reported having formal education regarding this population (Table 1). On a scale of 1–6, with higher numbers representing greater comfort, the mean comfort score interacting with people with IDD was 3.88 (CI 3.76–4.01) and the mean comfort score for treating people with IDD was 3.73 (CI 3.61–3.85), both of which correspond to between “a little uncomfortable” and “a little comfortable” (Table 1). Residents who trained in a pediatric-focused residency program reported having more formal education about persons with IDD, had more experience with this population, and were more comfortable with both interacting and treating this patient population when compared with their adult-focused resident counterparts (all *p* values <0.01) (Table 1).

**Table 1:** Demographic Information, Resident Experiences, and Resident Comfort Levels

	All residents	Adult-focused residents	Pediatric-focused residents	Difference in proportions or means (95% CI)	Cohen *d*	*p* Value^a^
Participants,^b^ n	423	293	127			
Mean age (range)	29.10 (23.00–38.00)	28.97 (24.00–38.00)	29.42 (23.00–38.00)			0.09
Sex, n (%)						0.01
Female	248 (60)	157 (55)	88 (71)			
Male	159 (38)	124 (43)	35 (28)			
Other	2 (1)	2 (1)	0 (0)			
Prefer not to answer	6 (1)	5 (2)	1 (1)			
Median PGY level	PGY-2	PGY-2	PGY-2			0.60
Treated at least 1 patient with IDD, n (%)	406 (96)	278 (95)	125 (98)	0.04 (−0.01 to 0.07)		0.09
Formal education dedicated to IDD, n (%)	104 (25)	29 (10)	74 (60)	0.50 (0.40 to 0.59)		<0.01
Mean amount of prior experience interacting with people with IDD (95% CI) [4-point scale, higher = more experience]	2.45 (2.38–2.52)	2.30 (2.23–2.38)	2.79 (2.65–2.92)	0.48 (0.33 to 0.63)	0.70	<0.01
Mean comfort level interacting with people with IDD (95% CI) [6-point scale, higher = more comfortable]	3.88 (3.76–4.01)	3.71 (3.56–3.85)	4.28 (4.06–4.51)	0.58 (0.31 to 0.84)	0.46	<0.01
Mean comfort level treating people with IDD (95% CI) [6-point scale, higher = more comfortable]	3.73 (3.61–3.85)	3.57 (3.44–3.71)	4.08 (3.86–4.30)	0.51 (0.25 to 0.77)	0.42	<0.01
Mean IDP scale score (95% CI) [lower score = less discomfort]	66.80 (65.74–67.86)	67.70 (66.49–68.92)	64.71 (62.59–66.83)	−2.99 (−5.43 to 0.55)	−0.27	0.02

Abbreviations: IDD = intellectual and/or developmental disability; IDP = Interactions with Disabled Persons; PGY = postgraduate year.

a *p* Values noted for differences between adult-focused and pediatric-focused residents; *t* test for continuous variables, χ^2^/Fischer exact test for categorical variables, and Wilcoxon test for the median PGY level.

b Residency specialty information was missing for 3 participants.

Pediatric-focused residents also had a lower mean IDP scale score, indicating less discomfort, compared with the adult-focused residents (64.71 vs 67.70, mean difference −2.99 [−5.43 to 0.55], *p* = 0.02).

Among respondents, 76% had treated a person with IDD as an inpatient, which was the most common experience reported (Table 2). Other common experiences were having a classmate with IDD before college (44%), treating a person with IDD as an outpatient (39%), and volunteering with an organization serving persons with IDD (26%) (Table 2). In Table 2, mean comfort scores are compared between residents with and without specific experiences regarding persons with IDD. With the exception of having a friend or college classmate with IDD which had very small sample sizes, all other experiences were associated with statistically significant higher mean comfort scores. The largest differences in mean comfort scores were for residents who were members of an organization that serves people with IDD (mean difference 0.73 [95% CI 0.44–1.01], *p* < 0.01, *d* = 0.53) and those with a family member with IDD (mean difference 0.55 [95% CI 0.23–0.88], *p* < 0.01, *d* = 0.44) (Table 2).

**Table 2:** Types of Experiences Residents Reported With Individuals With IDD

Type of experience	n (%)	Mean comfort level interacting with people with IDD (95% CI)	Difference in means (95% CI)	Cohen *d*	*p* Value
Treated an inpatient with IDD					
Yes	321 (76)	3.96 (3.82–4.10)	0.30 (0.58 to 0.02)	0.24	0.04
No	102 (24)	3.66 (3.41–3.90)			
Had a k-12 classmate with IDD					
Yes	186 (44)	4.08 (3.90–4.25)	0.34 (0.10 to 0.58)	0.27	0.01
No	237 (56)	3.73 (3.57–3.90)			
Treated an outpatient with IDD					
Yes	166 (39)	4.14 (3.94–4.33)	0.42 (0.17 to 0.67)	0.33	<0.01
No	257 (61)	3.72 (3.57–3.87)			
Member of an organization that serves people with IDD					
Yes	108 (26)	4.43 (4.18–4.68)	0.73 (0.44 to 1.01)	0.53	<0.01
No	315 (74)	3.70 (3.56–3.83)			
Has a family member with IDD					
Yes	73 (17)	4.34 (4.04–4.64)	0.55 (0.23 to 0.88)	0.44	<0.01
No	350 (83)	3.79 (3.66–3.92)			
Has a neighbor with IDD					
Yes	55 (13)	4.24 (3.88–4.59)	0.41 (0.03 to 0.78)	0.32	0.04
No	368 (87)	3.83 (3.70–3.96)			
A friend's family member has IDD					
Yes	49 (12)	4.22 (3.89–4.56)	0.38 (0.02 to 0.75)	0.31	0.04
No	374 (88)	3.84 (3.71–3.97)			
Knows a person with IDD in their religious community					
Yes	45 (11)	4.38 (4.04–4.72)	0.55 (0.19 to 0.92)	0.46	<0.01
No	378 (89)	3.83 (3.70–3.95)			
Has a friend with IDD					
Yes	42 (10)	4.14 (3.70–4.58)	0.29 (−0.17 to 0.74)	0.22	0.21
No	381 (90)	3.86 (3.73–3.98)			
Had a college classmate with IDD					
Yes	23 (5)	4.35 (3.79–4.91)	0.49 (−0.08 to 1.06)	0.38	0.09
No	400 (95)	3.86 (3.73–3.98)			

Abbreviation: IDD = intellectual and/or developmental disability.

### Predictors of Higher Comfort Levels

The relationship between comfort interacting and treating people with IDD and several candidate explanatory variables was explored with Spearman and partial Spearman correlation. In bivariate analysis, amount of experience with people with IDD had a moderate, positive association with higher comfort scores interacting and treating individuals with IDD (*r*_s_ = 0.45 [95% CI 0.37–0.53, *p* < 0.01], *r*_s_ = 0.42 [95% CI 0.33–0.50, *p* > 0.01], respectively) (Table 3). Training in a pediatric-focused residency specialty and number of hours of formal education about caring for people with IDD had a weak, positive association with higher comfort scores interacting with and treating individuals with IDD (Table 3). Older age had a weak, positive association with increased comfort treating individuals with IDD, but not with interacting with individuals with IDD (Table 3). PGY and sex were not correlated with the comfort level. Only amount of experience remained statistically significantly correlated with the comfort scores when other variables including training in a pediatric-focused residency program, amount of formal education about caring for patients with IDD, year of medical training, age, and sex were controlled for (Table 3, partial Spearman correlation coefficient). As part of a subgroup analysis, we explored this relationship in the adult-focused residents only, excluding pediatric-focused residents. The Spearman correlation for amount of prior experience with people with IDD and comfort interacting was 0.45 (95% CI 0.35–0.54, *p* < 0.001) and for comfort treating was 0.37 (95% CI 0.26–0.47, *p* < 0.001) (eTable 1, links.lww.com/NXG/A570).

**Table 3:** Spearman and Partial Spearman Correlations for Comfort Interacting With and Treating People With IDD

Variable	Comfort measure	Spearman correlation coefficient (95% CI)	*p* Value	Partial Spearman correlation coefficient^a^ (95% CI)	*p* Value
Amount of prior experience interacting with people with IDD [rated on 4-point Likert scale]	Comfort interacting	0.45 (0.37 to 0.53)	<0.01	0.42 (0.33 to 0.49)	<0.01
	Comfort treating	0.42 (0.33 to 0.50)	<001	0.38 (0.29 to 0.46)	<0.01
Pediatric-focused residency program	Comfort interacting	0.18 (0.09 to 0.28)	<0.01	0.03 (−0.07 to 0.13)	0.55
	Comfort treating	0.18 (0.08 to 0.28)	<0.01	0.06 (−0.04 to 0.16)	0.25
Amount of formal education about caring for patients with IDD	Comfort interacting	0.18 (0.08 to 0.28)	<0.01	0.04 (−0.06 to 0.14)	0.39
	Comfort treating	0.15 (0.05 to 0.25)	<0.01	−0.00 (−0.10 to 0.10)	0.97
Current year of medical training	Comfort interacting	0.05 (−0.05 to 0.14)	0.36	−0.03 (−0.13 to 0.07)	0.61
	Comfort treating	0.07 (−0.03 to 0.17)	0.16	0.00 (−0.10 to 0.10)	0.98
Age	Comfort interacting	0.08 (−0.02 to 0.18)	0.10	0.05 (−0.05 to 0.15)	0.33
	Comfort treating	0.12 (0.01 to 0.21)	0.03	0.08 (−0.02 to 0.18)	0.11
Sex	Comfort interacting	0.06 (−0.04 to 0.16)	0.23	0.09 (−0.01 to 0.19)	0.07
	Comfort treating	0.05 (−0.05 to 0.16)	0.29	0.09 (−0.01 to 0.19)	0.09

Abbreviation: IDD = intellectual and/or developmental disability.

a Partial Spearman correlation controls for all the other variables in the first column.

### Perceived Barriers

Using a 4-point scale, the highest-rated barrier to caring for patients with IDD was lack of knowledge about available resources to recommend (Table 4). When residents were asked how much different interventions would increase their comfort caring for patients with IDD, the highest rated intervention was having dedicated support staff to help navigate services for patients with IDD (Table 5).

**Table 4:** Barriers to Caring for Patients With IDD

Barrier	Mean (95% CI)
Lack of knowledge about available resources to recommend	3.22 (3.14–3.29)
Difficulty understanding the patient's level of functioning	2.77 (2.70–2.84)
Difficulty communicating with a person with IDD	2.69 (2.62–2.77)
The time it takes to see a patient with IDD	2.62 (2.53–2.70)
Lack of ability to control difficult behaviors	2.55 (2.46–2.64)
Lack of knowledge about childhood-onset conditions	2.53 (2.45–2.61)
The amount of paperwork involved	2.50 (2.41–2.59)
Difficulty obtaining details about care of their childhood-onset conditions	2.49 (2.41–2.56)
Difficulty with thorough physical examination because of physical barriers	2.43 (2.34–2.52)
Reliance on caregivers to provide medical history	2.40 (2.31–2.48)
Patients with IDD have many other doctors (i.e., specialists) involved in their care	2.34 (2.26–2.42)
Lack of comfort interacting with people with IDD	1.98 (1.90–2.06)
Clinic/hospital level concerns about lower reimbursement potential for patients with IDD	1.24 (1.18–1.30)

Abbreviation: IDD = intellectual and/or developmental disability.

Scale: 1 = not at all a barrier, 2 = a little bit of a barrier, 3 = somewhat of a barrier, 4 = very much a barrier.

**Table 5:** Interventions to Increase Resident Comfort Caring for Patients With IDD

Intervention	Mean (95% CI)
Dedicated support staff to navigate services for patients with IDD	3.74 (3.69–3.79)
Having a doctor who specializes in caring for patients with IDD in my clinic/department to talk through cases	3.49 (3.42–3.55)
More interactions with people with IDD	3.05 (2.98–3.13)
More didactic sessions/lectures about IDD	2.76 (2.68–2.84)
A dedicated rotation about care for patients with IDD	2.68 (2.58–2.77)
Online resource with facts and practice guidelines about patients with IDD	2.58 (2.49–2.67)

Abbreviation: IDD = intellectual and/or developmental disability.

Scale: 1 = not at all, 2 = a little, 3 = somewhat, 4 = very much.

## Discussion

Nearly all resident physicians care for adult or pediatric patients with IDD, but only a quarter of residents in our study reported that their residency training programs provided formal education regarding this patient population. Our a priori hypothesis that residents who trained in a pediatric-focused program would be more comfortable with this patient population compared with residents who trained in an adult-focused specialty was supported. However, the strongest predictor of increased comfort with this population was the amount of prior experience a resident had with individuals with IDD.

It is important to highlight the reported lack of formal education on this topic. Approximately 90% of residents in adult-focused specialties reported receiving no formal education about caring for people with IDD, which is comparable with a recent study that found only 11% of internal medicine and family medicine residents reported having formal education on this topic.^14^ Pediatric-focused residencies tended to have more education on this topic likely because intellectual and developmental disabilities have onset in childhood. However, as more children with IDD are living into adulthood and seeking care from adult-trained physicians, it is critically important to ensure that the workforce is equipped to provide equitable care to adult patients with IDD. In a national survey examining perceptions of physicians practicing in the United States, only two-fifths reported feeling “very confident” in their ability to provide equal care to people with disabilities.^21^ A lack of formal education about IDD among resident physicians may be a contributor to poorer health outcomes and higher mortality rates seen in people with IDD. Notably, when asked about ways to increase comfort in caring for patients with IDD, residents rated having more meaningful interactions with individuals with IDD higher than didactic sessions. Thus, the type of education that residents receive is also important to consider. Further investigation into the type and amount of education that residency programs provide to physician trainees is warranted.

Examining resident physicians' perceived barriers to caring for patients with IDD and ways to overcome these barriers addresses a critical education gap. In our study, resident physicians perceive the greatest barrier to be lack of knowledge about available resources to recommend. There are numerous online resources dedicated to caring for people with IDD.^22,23^ However, it is unknown if residents are aware of these resources or if they find them useful. Residency programs could distribute these online resources to resident physicians as a first step toward meeting their needs. In addition, having educational sessions specifically addressing resources for patients with IDD is necessary. There was a similar survey-based study examining primary care physicians' perceived barriers to caring for adults with childhood-onset chronic diseases (not specific to IDD), which found lack of time and reimbursement to be major barriers to caring for these patients.^10^ In our study, lack of time was perceived to be a barrier but concern about reimbursement was not. This difference is likely because trainees are inexperienced with billing and reimbursement rates do not directly affect them. In our study, the highest rated intervention for increasing resident comfort with individuals with IDD was having dedicated support staff to help navigate services for patients with IDD. Similarly, in the study among primary care physicians, improved care coordination was identified as a way to improve high-quality care for this patient population.^10^ These findings highlight the need for interdisciplinary teams in caring for this patient population and may be a valuable finding for organizations looking to advocate for additional support staff.

Although education and providing resources to residents are an important part of increasing their comfort level with this patient population, our study suggests that these interventions alone are likely not sufficient to reach this goal. Rather, our study supports increasing meaningful interactions between resident physicians and people with IDD to increase their comfort level. This is similar to results from a small pilot study demonstrating that family medicine residents who underwent experiential learning with patients with IDD in addition to didactic sessions reported significantly higher comfort levels with this patient population compared with baseline, while the residents who received only didactic sessions showed little to no improvement.^24^

In our study, we found the amount of prior experience with individuals with IDD greatly influenced overall comfort with this population. Residents in pediatric-focused training programs had more experience with people with IDD compared with residents in adult-focused training programs, which likely explains why the pediatric-focused residents were more comfortable interacting and treating patients with IDD compared with the adult-focused residents. In the subgroup analysis excluding pediatric-focused residents, the amount of experience with this patient population remained correlated with comfort levels, further supporting the notion that experience, rather than the specific type of training program, is the most important contributor to resident comfort caring for people with IDD. It is of interest that different types of experiences with people with IDD had different levels of association with overall resident comfort scores. The most common resident experience with people with IDD was caring for a person with IDD as an inpatient. However, this experience was not strongly associated with higher comfort scores. This is likely because the inpatient setting often lacks opportunities for finding connections or truly learning about the person admitted since time is limited, the focus is on providing acute care, and the patient is typically not their usual self. Being a part of an organization that serves persons with IDD corresponded to the most significant difference in comfort scores. It is possible that people who seek out this experience are already more comfortable with this population. Our findings also support that the closer and longer standing the relationship is with a person with IDD, the more comfortable they are with this population. These findings support contact theory, which proposes that increasing interactions with “dissimilar” people can lead to decreased negative attitudes toward that population.^25-28^ For example, we found that having a family member with IDD was associated with higher comfort scores compared with having a neighbor with IDD. Similarly, having a clinic patient with IDD was associated with higher comfort scores compared with an inpatient experience. This finding presents an opportunity to increase resident comfort with this patient population through enhanced outpatient exposure during training. Model clinics exist throughout the country that focus on the care of adults with childhood onset neurologic disorders, such as tuberous sclerosis and complex epilepsy syndromes. Medical educators should prioritize resident engagement in these unique clinical settings.

Limitations include the use of self-report surveys and the distribution of surveys only to residency programs in Boston, MA. Because convenience sampling was used, additional studies are needed to ensure generalizability. We also acknowledge the limitations of surveys in understanding attitudes and perceptions. Future research using qualitative methodology informed by our findings is needed. In addition, future studies should use more objective outcomes such as the number of patients with IDD residents care for in their clinic panel or specific health outcomes of those patients. It would also be useful to survey residency program directors on the intended and actual amount of disability-related education for their residents.

Prior experience with individuals with IDD predicted higher comfort levels with this population. This study supports the need for novel strategies to connect resident physicians to people with IDD to increase their comfort levels with this patient population and ultimately improve patients with IDD access to the high-quality health care that all patients deserve.

## Appendix Authors

**Table:**

Name	Location	Contribution
Hannah F. Johnson Shapiro, MD	Department of Neurology, Boston Children's Hospital, MA	Drafting/revision of the manuscript for content, including medical writing for content; major role in the acquisition of data; study concept or design; analysis or interpretation of data
Julia S. Frueh, MD	Department of Neurology, Boston Children's Hospital, MA	Drafting/revision of the manuscript for content, including medical writing for content; major role in the acquisition of data; study concept or design; analysis or interpretation of data
Madeline Chiujdea	Department of Neurology, Boston Children's Hospital, MA	Drafting/revision of the manuscript for content, including medical writing for content; major role in the acquisition of data; study concept or design; analysis or interpretation of data
Stefan Sillau, PhD	Department of Neurology, University of Colorado School of Medicine, Aurora	Drafting/revision of the manuscript for content, including medical writing for content; analysis or interpretation of data
Jessica Solomon Sanders, MD	Department of Neurology, and Department of Pediatrics, University of Colorado School of Medicine, Aurora	Drafting/revision of the manuscript for content, including medical writing for content; major role in the acquisition of data; study concept or design; analysis or interpretation of data

## Glossary

IDD: intellectual and/or developmental disability
IDP: Interaction with Disabled Persons
PGY: postgraduate year
REDCap: Research Electronic Data Capture
